# Study of serum and urinary markers of the reninangiotensin-aldosterone system in myelomeningocele patients with renal injury detected by DMSA

**DOI:** 10.1590/S1677-5538.IBJU.2019.0797

**Published:** 2020-07-31

**Authors:** Cássia Maria Carvalho Abrantes do Amaral, Dulce Elena Casarini, Maria Cristina Andrade, Marcela Leal da Cruz, Antônio Macedo

**Affiliations:** 1 Universidade Federal de São Paulo - UNIFESP Departamento de Pediatria São Paulo SP Brasil Departamento de Pediatria, Universidade Federal de São Paulo - UNIFESP, São Paulo, SP, Brasil; 2 Centro de Apoio à Criança com Anomalia Urológica -CACAU Departamento de Urologia São Paulo SP Brasil Departamento de Urologia, Centro de Apoio à Criança com Anomalia Urológica -CACAU - Núcleo de Urologia Pediátrica - NUPEP, São Paulo, SP, Brasil; Núcleo de Urologia Pediátrica - NUPEP Brasil

**Keywords:** Meningomyelocele, Acute Kidney Injury, Renin-Angiotensin System

## Abstract

**Introduction:**

The Renin-Angiotensin-Aldosterone System (RAAS) has been suggested as a possible marker of renal injury in chronic diseases. This study proposes to analyze the serum and urinary markers of the RAAS in myelomeningocele patients with renal function abnormalities detected on DMSA.

**Material and Methods:**

Seventeen patients followed in our institution that presented with renal injury on DMSA. We review nephrologic and urologic clinical aspects and evaluated ultrassonagraphy, voiding urethrocystography and urodynamics. Urinary and serum samples were collected to evaluate possible correlations of renal lesions with RAAS. Control group urine and serum samples were also sent for analysis.

**Results:**

Serum ACE 2 activity means in relation to urodynamic findings were the only values that had a statistically significant difference (p = 0.040). Patients with normal bladder pattern presented higher ACE 2 levels than the high risk group. Statistical analysis showed that the study group (SG) had a significantly higher mean serum ACE than the CG. The means of ACE 2 and urinary ACE of the SG and CG were not statistically different. The ROC curve for serum ACE values had a statistically significant area for case and non-case differentiation, with 100% sensitivity and 53% specificity for values above 60.2 mg/dL. No statistically significant areas were observed in relation to ACE 2 and urinary ACE values between SG and CG.

**Conclusion:**

The analysis of serum ACE, ACE 2 and urinary ACE were not significant in patients with myelomeningocele and neurogenic bladder with renal injury previously detected by renal DMSA.

## INTRODUCTION

Myelomeningocele (MMC) is the main pathology associated with neural tube closure defects with an incidence of 1.9 to 3.7 per 10.000 live births (1, 2). It is known that the neurogenic bladder due to this anomaly requires follow-up from birth because of the risk of renal deterioration.

The Renin-Angiotensin-Aldosterone System (RAAS) plays an important role in regulating blood pressure and electrolyte homeostasis through the release of renin by juxtaglomerular cells, and it has been suggested as a possible marker of renal injury in chronic diseases.

Gobet et al. (3) observed that in fetal-onset renal diseases, as well as in postnatal renal diseases, RAAS played an important role in renal interstitial fibrosis, possibly by activating the transforming growth factor-beta (TGF-**β**1), which has the function of controlling cell proliferation and differentiation, and other functions in most cells.

This study proposes to analyze the serum and urinary markers of the Renin-Angiotensin-Aldosterone System in myelomeningocele patients with renal function abnormalities detected on DMSA scintigraphy in order to identify whether they can be used as diagnostic and prognostic markers of the kidney damage secondary to bladder abnormalities that may occur in this condition.

## MATERIALS AND METHODS

The research was registered and approved by the Research Ethics Committee (REC) of our institution. Eighty-seven patients undergoing regular follow-up were studied at Pediatric Nephrology Outpatient Clinic in our institution. From these, 21 (24%) presented a description in the DMSA renal scintigraphy alteration chart. A blinded reassessment of the examinations was then performed by a specialist in Nuclear Medicine, confirming the change in 17 patients representing the study group (SG). The control group (CG) consists of age-matched patients without urological diseases.

These patients were summoned to a new interview with detailed review of the following parameters: gender, age, antenatal data, voiding habit, intermittent catheterization, use of medications, ventricular-peritoneal shunt, evolution to renal disease (through dosing of serum and urinary urea and creatinine), presence of vesicoureteral reflux, presence of hydronephrosis and bladder thickening, presence of chronic intestinal constipation (Bristol Scale) (4), number of urinary tract infections in the last year, evaluation of systemic blood pressure, evaluation of weight and height according to age and gender.

The presence of pyelonephritis (febrile episode with urine I leukocyte alteration and positive urine culture above 100.000cfu/mL), as well as the need for hospitalization were reported at the initial assessment, and at each visit to the Nephropediatric Outpatient Clinic.

Patients were classified according to the stage of renal injury they presented, using the estimated glomerular filtration rate (eGFR) based on the Schwartz equation (2012) updated (5). Patients with eGFR above 90mL/min/1.73m2 were considered without renal function impairment and those with eGFR below 90mL/min/1.73m^2^ with alteration.

Technetium-99m-labeled DMSA static renal scintigraphy (99mTc-DMSA) was evaluated according to a study proposed by Ono et al., (6) and classified as: renal uptake greater than 45%: absence of renal injury, renal uptake between 44 to 40%: mild injury, renal uptake between 39 and 35%: moderate injury and renal uptake between 34 and 30%: severe injury.

The renal and urinary tract ultrasound were evaluated for the presence of hydronephrosis and thickening of the bladder walls. Hydronephrosis was classified from grades 1 to 4 according to the classification of the Fetal Urological Society (7). The bladder wall was named normal or hypertrophic (above 3mm) (8). The presence of VUR was assessed by voiding urethro-cystography and classified according to the classification of the International Reflux Study (IRS 1-5) (9).

The urodynamic study (UDS) allowed the update of the bladder pattern classification distributed in the following groups (10): normal pattern, high risk pattern: Patients with Detrusor Leak Point Pressure (DLPP) from 40cm H2O, high detrusor pressure during bladder filling or hyperactivity amplitude from 40cm H2O, incontinent pattern and hypocontractile pattern.

Urinary and serum samples were collected from 17 selected patients to evaluate possible correlations of renal lesions with RAAS. CG urine and serum samples were also sent for analysis to quantify serum Angiotensin-Converting Enzyme (ACE) and Angiotensin-Converting Enzyme 2 (ACE 2) and urinary ACE activities. The urine collected samples were immediately frozen and individually processed after measurement of their volume and pH correction. There was adjustment of pH to 8.0 with Tris buffer 1M and the urine submitted to centrifuge (3000rpm). ACE activity was determined fluorimetrically using Z-Phe-His-Leu (Z-Phe-HL) as substrate (11). ACE 2 activity in serum was fluorimetrically determined based on the work of Pedersen et al. (12). Sample assays were performed in duplicate, with intrinsic fluorescence corrected by white.

For all statistical tests, a significance level of 5% was used. Statistical analyzes consisted of the Chi-square test, or alternatively in small sample cases, Fisher’s exact test. Comparison of means between two independent groups and between two related samples (RG and CG paired by gender and age) were performed respectively by Student’s t-tests for independent and paired samples.

Comparison of means between more than two independent groups was performed using the non-parametric Kruskal-Wallis test due to the sample 2 size. At detecting mean differences, the identification of groups with distinct means was performed via Dunn-Bonferroni multiple comparisons to maintain the global significance level.

The ROC curve was used to assess the discriminatory capacity of cases and non-cases of altered DMSA, according to the RAAS activity indicators. Areas under the curve are considered significative in accordance with the p-value <0.05.

## RESULTS

Information from 87 patients whose mean age was 8.1 years (SD=6.9 years and range from 4 months to 24 years) was analyzed. From the 87 patients evaluated, 17 (19.5%) had altered DMSA.

The clinical characteristics of the patients studied with DMSA scintigraphy are shown in Table-1. There is a predominance of females (82.4%). It is also noted that almost half of the SG was diagnosed before birth (intrauterine) and 7 out of 10 started treatment at birth. The average gestational age (GA) at birth was 38.2 weeks (SD=1.4 weeks), ranging from 36 to 41 weeks.

**Table 1:** Clinical aspects in Study Group (SG).

		Average	SD
**Gestational Age (weeks)**		38.2	1.4
		N	%
**Gender**		17	100.0
	Female	14	82.4
	Male	3	17.6
**Mobility**			
	Good	6	35.3
	Regular	5	29.4
	Bad	6	35.3
**Comorbidity**			
	Yes (Arterial Hypertension)	1	5.9
	No	16	94.1
**IMC (kg/m^2^)**			
	Eutrophy	11	64.7
	Overweight	4	23.5
	Obesity	2	11.7
**Level of Medular Lesion**			
	Lumbar	7	41.2
	Sacral	7	41.2
	Thoracic	5	29.4
**Hydrocephalus**			
	No	2	11,8
	Yes	15	88,2
**Moment at diagnosis**			
	Postnatal	8	47.1
	Prenatal	9	52.9
**Moment at beginning of urological treatment**			
	At birth	14	82.4
	After 1 year old	3	17.6
**Clean Intermittent Catheterization (CIC)**			
	No	1	5.9
	Yes	15	88.2
	Vesicostomy	1	5.9
**Person who peforms CIC**			
	Mother	9	56.3
	Patient	7	43.8
**Anticholinergics**			
	No	9	52.9
	Yes	8	47.1
**Antibiotic Prophylaxis**			
	No	7	41.2
	Yes	10	58.8
**UTI**			
	No	3	17.6
	Yes	14	82.4
**Renal Function**			
	Abnormal	4	23.5
	Normal	13	76.5
**Bristol Score**			
	1	16	94.1
	5	1	5.9

In regards to mobility, 35.3% of the SG had good mobility, 29.4% regular and 35.3% bad. It was also observed that 94.1% had no comorbidities, 41.2% presented lumbar injury level and 41.2% sacral level, 88.2% of patients with hydrocephalus and need of VPS in 93.3% of cases. In addition, 94.1% of patients had Bristol score grade 1 and 88.2% were submitted to intermittent bladder catheterization, and in 56.3% of cases, the mother was responsible for performing the procedure. Also regarding urological treatment, 47.1% were on anticholinergics and 58.8% were on antibiotic prophylaxis. The presence of UTI was observed in 82.4% of patients and 23.5% had impaired renal function.

Regarding the findings of nephro-urological imaging exams and urodynamic features of the SG, it was observed the presence of pyelocalix dilation on USRV in 94.1% of the patients, and in 47.1% the dilation was only pelvic with normal renal parenchyma. In addition, 82.4% of SG had mild or moderate DMSA lesions (similar distribution of mild and moderate lesions). Additionally, 52.9% presented vesicoureteral reflux. The UDS was changed in 76.4%, with 52.9% of patients with incontinent pattern and 23.5% with high risk pattern.

Table-2 shows the activities of serum ACE and ACE 2, and urinary ACE. Serum ACE activity in SG did not show significant changes according to the variables evaluated.

**Table 2:** Serum ACE, ACE 2 and urinary ACE activity in Study Group.

		Average (SD)		Median	n	p
**Serum ACE (nmol/mL/min.)**						
**DMSA**						**0.282**
	44 to 40 %: Mild lesion	93.81 (27.88)		90.7	7	
	39 to 35%: Moderate lesion	116.94 (38.72)		103.6	7	
	34 to 30%: Severe lesion	112.73 (9.95)		117.5	3	
**VCUG - VUR grade**						**0.923** a
	0	108.6 (37.0)		100.3	8	
	1	119.6 (16.70)		118.4	6	
	2	74.4 (18.0)		68.1	3	
**UDS - Bladder Pattern**						**0.187**
	Normal	87.53 (23.70)		86.1	4	
	Incontinent	110.10 (38.12)		99.3	9	
	High Risk	118.13 (12.30)		117.1	4	
**UTI**						0.365
	No	91.20 (9.85)		90.7	3	
	Yes	109.90 (33.70)		109.8	14	
**Creatinine Clearance Stage**						**0.652**
	1	123.53 (24.32)		101.3	13	
	2	116.87 (52.33)		111.1	4	
**ACE 2 (μmol/min./mL)**						
	**DMSA**					**0.269**
	44 to 40 %: Mild lesion	0.19 (0.15)		0.16	7	
	39 to 35%: Moderate lesion	0.14 (0.16)		0.07	7	
	34 to 30%: Severe lesion	0.17 (0.07)		0.2	3	
**VCUG - VUR grade**						**0.700** a
	0	0.16 (0.14)		0.13	8	
	1	0.16 (0.16)		0.08	6	
	2	0.17 (0.10)		0.17	3	
**UDS - Bladder Pattern**						**0.040**
	Normal	0.29* (0.14)		0.25	4	
	Incontinent	0.15 (0.13)		0.1	9	
	High Risk	0.07*	0.07	4		
		(0.02)				
**UTI**					**0.097**	
	No	0.28	0.20	3		
		(0.17)				
	Yes	0.13	0.08	14		
		(0.12)				
**Creatinine Clearance Stage**					**0.391**	
	1	0.18	0.10	13		
		(0.15)				
	2	0.11	0.11	4		
		(0.06)				
**Urynary ACE (mg/mL) /creatinine**						
	**DMSA**				**0.550**	
	44 to 40 %: Mild lesion	0.45	0.40	7			
		(0.26)				
	39 to 35%: Moderate lesion	0.44	0.50	7		
		(0.15)				
	34 to 30%: Severe lesion	0.63	0.60	3		
		(0.45)				
**VCUG - VUR grade**					**0.680**	
	0	0.52	0.35	8		
		(0.32)				
	1	0.48	0.30	6		
		(0.21)				
	2	0.36	0.55	3		
		(0.11)				
**UDS - Bladder Pattern**					**0.590**	
	Normal	0.42	0.35	4		
		(0.26)				
	Incontinent	0.45	0.30	9		
		(0.29)				
	High Risk	0.60	0.55	4		
		(0.14)				
**UTI**					**0.053**	
	No	0.76	0.80	3		
		(0.35)				
	Yes	0.42	0.40	14		
		(0.19)				
**Creatinine Clearance Stage**					**0.080**	
	1	0.42	0.40	13		
		(0.18)				
	2	0.67	0.70	4		
		(0.37)				

**ACE =** Angiotensin-Converting Enzyme 1; **ACE 2 =** Angiotensin-Converting Enzyme 2; **DMSA =** Dimercaptosuccinic Acid Scintigraphy; **UDS =** Urodynamic Study; **UTI =** Urinary Traction Infection; **VCUR =** Voiding Cystourethrogram; **VUR =** Vesicoureteral Reflux

* = Significant differences according to multiple Dunn-Bonferroni comparisons.

a = p = Descriptive level of the Kruskal-Wallis or Mann-Whitney test

Serum ACE 2 activity means in relation to UDS findings were the only values that had a statistically significant difference (p=0.040) (Table-2). Patients with normal UDS classification presented higher ACE 2 levels than the high risk group. There were no differences in ACE 2 in the incontinent group compared to the other groups. Regarding the association between the ACE, ACE 2 and urinary ACE dosages and the patient’s creatinine clearance stage, no statistical differences were observed.

Statistical analysis comparing the results of serum ACE, ACE 2 and urinary ACE between the study (SG) and control (CG) groups showed that the SG had a significantly higher mean serum ACE (106.68nmol/mL/min) than the CG (74.06nmoL/ mL/min). The means of ACE 2 and urinary ACE of the patient and control groups were not statistically different.

**Figure 1:**
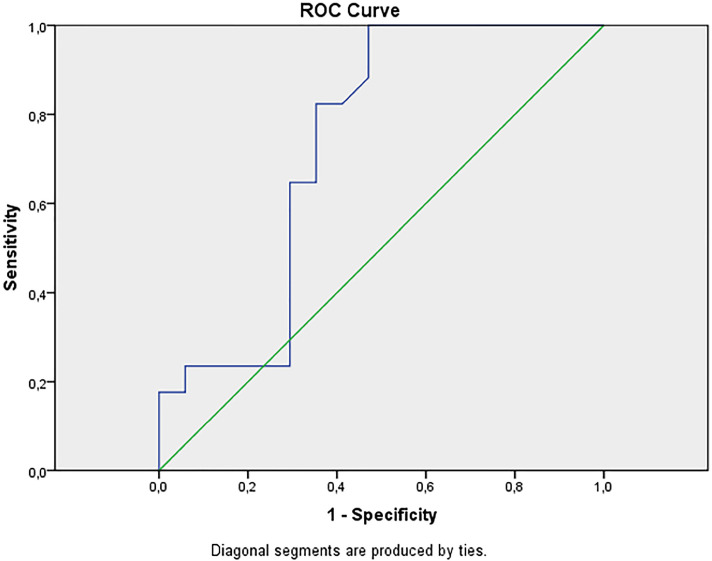
ROC curve for serum ACE values in the Study and Control Groups.

According to Figure-1, the ROC curve values for serum ACE values had a statistically significant area for case and non-case differentiation, with 100% sensitivity and 53% specificity for serum ACE values above 60.2mg/dL. No statistically significant areas were observed in relation to ACE 2 and urinary ACE values between SG and CG (sensitivity and specificity).

## DISCUSSION

In this study, it was observed that the vast majority of SG started their treatment in the first year of life (82.4%), but about half of them (47.1%) had their diagnosis only at birth. These results show us a prenatal pattern with some difficulties, since the intrauterine diagnosis of these patients would help both in terms of procedures and in the follow-up of these cases even earlier.

Only 3 of 17 children (17.6%) with abnormal DMSA started treatment after 1 year of life. Bauer in 2008 (12) observed that the institution of CIC and anticholinergic therapy in childhood revealed many advantages over time, as it leads to the prevention of the development of renal damage, as well as allows the patient autonomy in relation to its treatment. In a paper conducted by Elzeneini et al. (14), the authors observed that the performance of CIC in the first years of life is a prevention factor for renal damage, especially in women, which was not observed in this study, since of the 17 patients with altered DMSA, about 80% of the group consisted of women.

Lehnert et al. (15) and Humblet et al. (16) observed that patients who presented high detrusor pressure associated with low bladder emptying in urodynamic studies had renal damage and recurrent urinary tract infection with potential for the development of chronic renal disease and systemic arterial hypertension. In the study, about 75% of children with abnormal DMSA had urodynamic study abnormalities, with bladders associated with low bladder compliance or of high pressure, but with normal blood pressure levels.

Around up to one third of children with neurogenic bladder have VUR. (17, 18) VUR in these patients is usually secondary, and its main causes are associated with increased intravesical pressure (19, 20) and may also be secondary to recurrent urinary tract infections (which may cause weakening of the bladder valve mechanism), in addition to the dysfunctional urination process perpetuating the VUR in these patients (21). In this study, we observed the presence of VUR in about 50% of patients with renal injury diagnosed with DMSA, 23.5% of them with grade III and 17.7% with grades IV or V.

Follow-up of patients with MMC should be monitored by USRV, but in patients with obesity or scoliosis, DMSA should always be performed (22). The presence of renal scars increases the risk of hypertension and worsens renal injury. In this study, it was observed that the alteration of USRV was more evident in patients with altered DMSA (70.6%), with confirmed alteration of USRV in 94.1% of RG, while 47.1% of patients without altered DMSA also had altered USRV.

The renin-angiotensin-aldosterone system (RAAS) is currently one of the main involved in the mechanism of pressure reduction and renoprotection with several randomized controlled studies showing the reno-protective potential of angiotensin-converting enzyme inhibitors (ACE inhibitors) and angiotensin II (ARB) in nephropathies of almost any etiology.

The optimal dose of angiotensin-converting enzyme inhibitors (ACEI) or angiotensin II receptor antagonists (ARA II) reduces albuminuria or proteinuria and decreases the development of renal dysfunction more than placebo. However, there are no clinical evidences whether these strategies may influence long-term renal prognosis (23).

Recent studies suggest that urine protein composition could be a good information tool for pathogenic renal mechanisms, which would be of great importance in establishing the appropriate treatment for each pathophysiological mechanism (24-28). Despite the patients evaluated in this study already present renal damage detected in DMSA, there was no relationship between increased urinary ACE and altered creatinine clearance, nor its association with different stages of DMSA.

When relating serum ACE, ACE 2 and urinary ACE to radiological, urodynamic parameters and creatinine clearance stage, only ACE 2 was altered, with statistical significance when comparing the normal urodynamic study in relation to the high risk altered. However, this analysis was made in only 4 cases in each group and therefore, has a very limited value.

Despite advances in the understanding of RAAS as a participant in the mechanism of renal injury, the analysis of its serum (ACE and ACE 2) and urinary (urinary ACE) markers did not present significance in the diagnosis associated with the nephron-urological characteristics of patients with MMC and NB with renal injury previously detected by renal DMSA scintigraphy. We can conclude that only serum ACE was statistically significant to identify patients with renal injury. Nevertheless, it is worth remembering that the study sample had already departed from the diagnosis of renal injury by means of renal DMSA scintigraphy.

As limiting factors of the study we can highlight a small number of patients that prevents the generalization of the results. Data were obtained from a highly complex outpatient clinic in a tertiary sector, which may not represent the general patient population. On the other hand, as strengths of this research, the methodology used was standardized and the exams were evaluated by specialists. The patient’s sample was homogeneous in relation to the clinical conditions and the control group was paired in accordance with gender and age.

## CONCLUSIONS

Despite advances in the understanding of RAAS as a participant in the mechanism of renal injury, the analysis of its serum (ACE and ACE 2) and urinary (urinary ACE) markers were not significant in patients with MMC and BN with renal injury previously detected by renal DMSA scintigraphy.
